# Deep learning based identification of bone scintigraphies containing metastatic bone disease foci

**DOI:** 10.1186/s40644-023-00524-3

**Published:** 2023-01-25

**Authors:** Abdalla Ibrahim, Akshayaa Vaidyanathan, Sergey Primakov, Flore Belmans, Fabio Bottari, Turkey Refaee, Pierre Lovinfosse, Alexandre Jadoul, Celine Derwael, Fabian Hertel, Henry C. Woodruff, Helle D. Zacho, Sean Walsh, Wim Vos, Mariaelena Occhipinti, François-Xavier Hanin, Philippe Lambin, Felix M. Mottaghy, Roland Hustinx

**Affiliations:** 1 The D-Lab, Department of Precision Medicine, GROW- School for Oncology and Developmental Biology, Maastricht University, Maastricht, The Netherlands; 2 Department of Radiology and Nuclear Medicine, Columbia University Irving Medical Center, New York, United States; 3 Division of Nuclear Medicine and Oncological Imaging, Department of Medical Physics, University Hospital of Liege, Liege, Belgium; 4 Department of Nuclear Medicine and Comprehensive diagnostic centre Aachen (CDCA), University Hospital RWTH Aachen University, Aachen, Germany; 5 Radiomics (Oncoradiomics SA), Liege, Belgium; 6 Department of Diagnostic Radiology, Faculty of Applied Medical Sciences, Jazan University, Jazan, Saudi Arabia; 7 Department of Nuclear Medicine, Clinical Cancer Research Centre, Aalborg University Hospital, Aalborg, Denmark; 8 Department of Clinical Medicine, Aalborg University, Aalborg, Denmark; 9 Department of Nuclear Medicine, Universite´CatholiqueUniversite´Catholique de Louvain, CHU-UCL-Namur, Ottignies-Louvain-la-Neuve, Belgium

**Keywords:** Deep learning, Metastatic bone disease, Bone scintigraphy, Activation maps

## Abstract

**Purpose:**

Metastatic bone disease (MBD) is the most common form of metastases, most frequently deriving from prostate cancer. MBD is screened with bone scintigraphy (BS), which have high sensitivity but low specificity for the diagnosis of MBD, often requiring further investigations. Deep learning (DL) - a machine learning technique designed to mimic human neuronal interactions- has shown promise in the field of medical imaging analysis for different purposes, including segmentation and classification of lesions. In this study, we aim to develop a DL algorithm that can classify areas of increased uptake on bone scintigraphy scans.

**Methods:**

We collected 2365 BS from three European medical centres. The model was trained and validated on 1203 and 164 BS scans respectively. Furthermore we evaluated its performance on an external testing set composed of 998 BS scans. We further aimed to enhance the explainability of our developed algorithm, using activation maps. We compared the performance of our algorithm to that of 6 nuclear medicine physicians.

**Results:**

The developed DL based algorithm is able to detect MBD on BSs, with high specificity and sensitivity (0.80 and 0.82 respectively on the external test set), in a shorter time compared to the nuclear medicine physicians (2.5 min for AI and 30 min for nuclear medicine physicians to classify 134 BSs). Further prospective validation is required before the algorithm can be used in the clinic.

**Supplementary Information:**

The online version contains supplementary material available at 10.1186/s40644-023-00524-3.

## Background

Metastatic bone disease (MBD) is the most common form of metastatic lesions [1, 2]. The incidence of bone metastasis varies depending on the cancer type [3], yet around 80% of MBD arise from breast and prostate cancers [4]. MBD, as the name implies, is due to the propensity of these tumours to metastasize to bones, and it results in eventually difficulty treating painful lesions. Henceforth, early diagnosis is necessary for individualized management that could significantly improve a patient’s quality of life [5].

MBD is usually detected using radionuclide bone scintigraphy (or bone scans, BS). BS are nuclear medicine images, which are used frequently to evaluate the distribution of active bone formation, related to benign or malignant processes, in addition to physiological processes. BS scans are indicated in a spectrum of clinical scenarios including exploring unexplained symptoms, diagnosing a specific bone disease or trauma, and the metabolic assessment of patients prior to and during the treatment [6, 7]. BS combining whole-body planar images and tomographic acquisition (SPECT – single photon emission computed tomography) on selected body parts are highly sensitive, as they detect metabolic changes earlier than conventional radiologic images, with lower sensitivity to lytic lesions. However, depending on the pattern it may lack the specificity to identify the underlying causes. Therefore, a SPECT/CT that correlates the findings of bone scintigraphy anatomically is often useful and leads to a more specific diagnosis of the changes noted [8], although MRI scans may also be additionally requested to clarify the diagnosis. Hence, a tool to improve the specificity of decisions based on BS, and reduce the need for further imaging is a relevant unmet clinical need.

Deep learning (DL) is a branch of machine learning (ML), and refers to data driven modelling techniques, which applies the principles of simplified neuron interactions [9]. The application of imaging analysis techniques using artificial neurons on medical imaging started to draw attention decades ago [10], but it only became a major research focus recently due to the advancement in computational capacities and imaging techniques [11, 12]. The artificial neuron model is used as a foundation unit to create complex chains of interactions - DL layers. These layers are used to generate even more complex structures - DL architectures. The neural network (NN) training procedure is typically a cost-function minimization process. The cost function measures the error of predictions based on the ground truth labels [13], and the DL network learns how to solve a problem directly from existing data, and apply it to data it has never seen. These complex models contain the parameters (weights) for millions of neurons, which can be trained for the recognition of problem-related patterns in the data being analysed.

Several studies investigated the potential of DL-based algorithms for analysing bone scintigraphy scans [14–16]. The majority of these studies applied DL-algorithms on BS scans of diagnosed (specific) cancer patients, which could limit the learning ability of the DL-algorithm to differentiate MBD from other bone diseases.

In this study, we hypothesize that DL-based algorithms can learn the pattern of metastatic bone disease on bone scintigraphy scans, and differentiate it from other non-metastatic bone diseases. We investigate the potential of a DL-based algorithm to detect MBD on BS, not limited to those of cancer patients, based on activation maps obtained using the gradient weighted class activation mapping (Grad-CAM) method [17, 18]. By doing so, we aim to develop a generalizable tool that can classify scans containing metastases and detect MBD on BS. Moreover, extracting activation maps with the Grad-CAM method [19] and superimposing these maps to the original BD scans, we explored the explainability of the deep learning model’s predictions. This is very important to promote the application of these methods in the clinic and avoid the common misconception that sees DL models as “black boxes” without any real connection to clinical and imaging characteristics.

## Methods

### Imaging data

The imaging data were retrospectively collected from different European centres: Aachen RWTH University Clinic (Aachen, Germany), Aalborg University Hospital (Aalborg, Denmark), and Namur University Hospital (Namur, Belgium). The scans were acquired at each center, following local protocols and with different scanner and acquisition parameters. The electronic medical records of these hospitals were searched for patients who underwent BS between 2010 and 2018. Patients for whom a definitive classification of the foci was available, mostly through further investigations, were further included. All images were acquired with anteroposterior (AP) and posteroanterior (PA) whole-body views. The imaging analysis was approved by the Aachen RWTH institutional review board (No. EK 260/19). According to Danish National Legislation, the Danish Patient Safety Authority can waive informed consent for retrospective studies (approval 31-1521-110). All methods were carried out in accordance with the relevant guidelines and regulations [20]. The study protocol for the in silico trial was published on clinicaltrials.gov (NCT: NCT05110430). Manual segmentation of the metastatic spots was performed on 25 BS scans coming from Namur University Hospital by the treating radiation oncologists.

### Image pre-processing

**Fig. 1:**
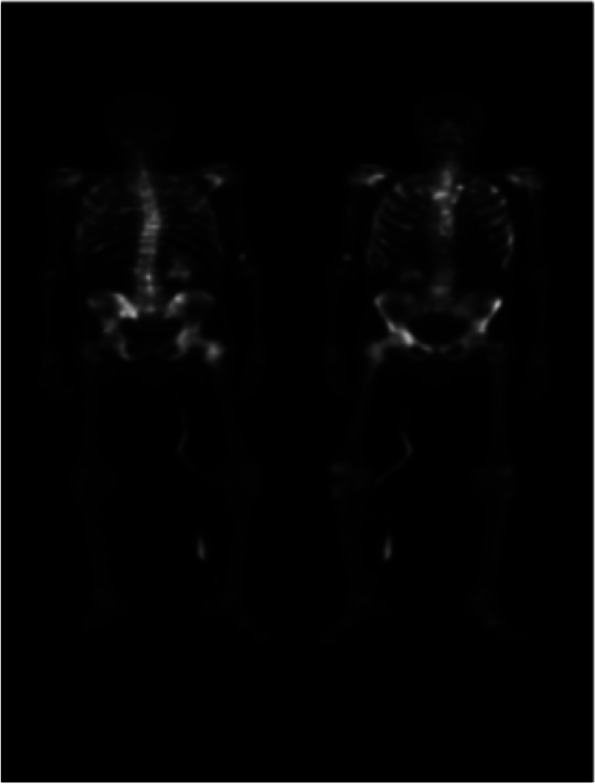
Example of pre-processed BS scans used as input for model training

Every datapoint containing acquisition at two views (AP and PA) was resized to size (length = 256, height = 512) and the intensities were normalized to range [0–1] using the minimum and maximum intensity of each image. For all the data points, image acquisitions at both views are appended besides each other as shown in Fig. 1.

### Model architecture, training and testing

The training and validation datasets are composed of 1203 and 164 images respectively, coming from Centre A (Aachen) and B (Aalborg). The external test cohort is composed of 998 images collected at centre C (Namur). A full overview of the patients cohort division between the different datasets is reported in Table 1.

**Table 1:** Division of the patients cohort between training, validation and external test

	Training (*n* = 1203)	Validation (*n* = 164)	External test (*n* = 998)
Centre A (Aachen)	235 with metastasis 668 normal	58 with metastasis 58 normal	-
Centre B (Alborg)	94 with metastasis 206 normal	24 with metastasis 24 normal	-
Centre C (Namur)	-	-	411 with metastasis 587 normal

The model was trained on 329 images containing metastasis from Centre B (94) and A (235). At each epoch, the 874 images without any metastasis were shuffled and 329 images were randomly selected to train the model with balanced labels. VGG16 architecture with ImageNet pretrained weights [21] was trained with categorical cross entropy loss for 6 epochs with 200 steps per epoch. The model was trained with 3 channel input. The pre-processed input was duplicated in all the channels, concatenating the inputs along the whole channels dimension to match the size of the pretrained ImageNet. During the training, the images were augmented [22] by flipping along the vertical axis so that the views at AP and PA were randomly represented in the left or right in the images.

**Fig. 2:**
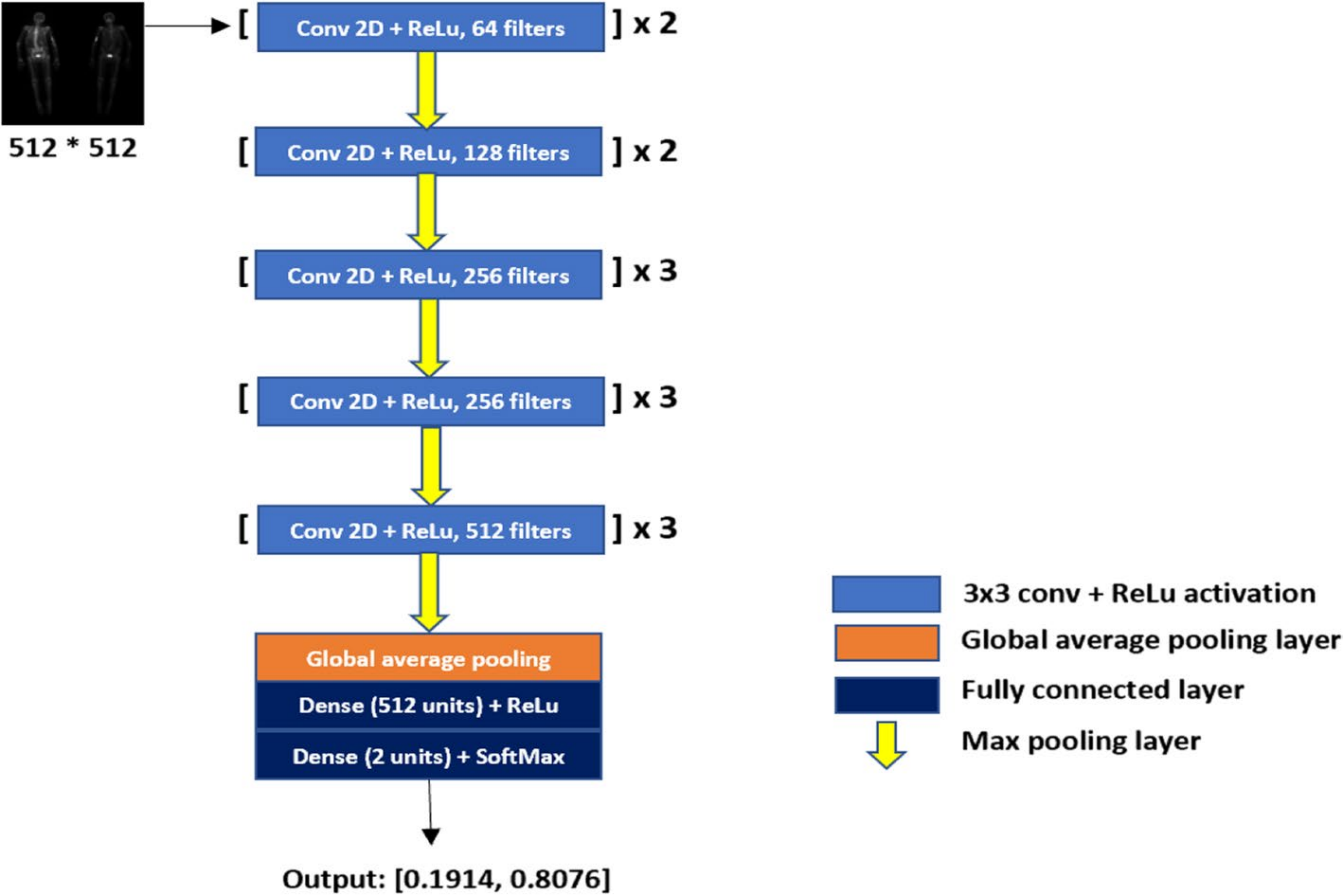
The architecture used in the study. Pre-processed BS scans resized to 512 * 512 dimensions were provided as input to the network. The network outputs a probability score for presence and absence of metastasis on BS images. X = block repetitions, Conv = Convolution kernel, ReLU = rectified linear unit, 3 × 3 = the size of the 2D CNN kernels

The last Max Pooling layer in the VGG16 model was followed by a Global Average pooling layer, followed by a fully connected layer with 512 units and ReLu activation, which is followed by a classification layer containing 2 units with Softmax activation [23] as shown in Fig. 2. The network weights are updated by using the Adam optimizer at learning rate of 1e^− 4^ [24]. The trained model’s performance was evaluated on an external test dataset (*n* = 998).

The following software packages were used: Python v3.6, Keras v2.0.6 for modelling, training and validation and Sklearn v1.1.1 for metrics calculation and results visualization. The model was trained and validated on a 11GB NVidia GeForce GPU.

### Quantitative metrics

The quantitative model performance in this study was assessed using ROC AUC, sensitivity and specificity of the classifier and confusion matrix (true positive rate (TPR), true negative rate (TNR), false negative rate (FNR) and false positive rate (FPR)). The model was evaluated according to the Checklist for AI in Medical Imaging (CLAIM) [25] and Standards for Reporting Diagnostic accuracy studies (STARD) [26].

### In silico clinical trial

**Fig. 3:**
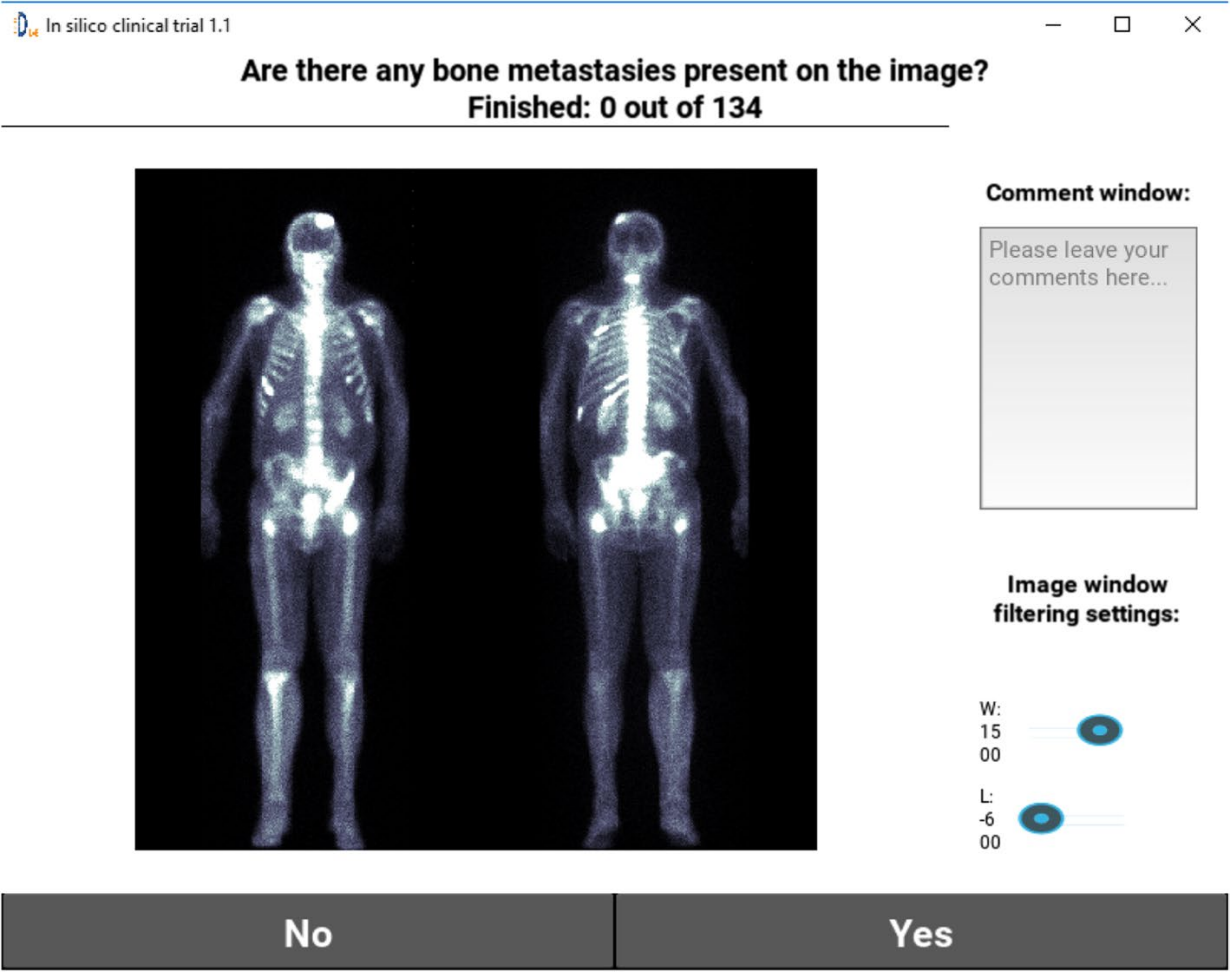
Screenshot of the application feedback window used in the in silico trial

To better gauge the proposed DL model performance, we developed an application allowing the creation of a reference performance point by collecting nuclear medicine physician’s feedback based on the visual assessment of BS scans. We have enrolled 6 nuclear medicine physicians (from one to ten years’ experience) to measure their performance on the evaluation dataset of 134 BS images. This dataset was sampled from the Centre C images with an equal number of negative and positive cases. In order to collect participant’s feedback, the application was displaying BS image, comment window and window filtering settings (Fig. 3). In the end of the feedback assessment an excel file was generated. For better visual comparison we have evaluated DL based AUC on the same dataset that has been used for visual assessment (134 BS images). Bootstrapping technique, involving 100 resamples obtained via random sampling with replacement from the same dataset, was utilized to estimate ROC AUC 95% confidence interval. Also F1 scores have been calculated and reported for the performance of both the model and the reader study.

## Results

### Model performance

**Fig. 4:**
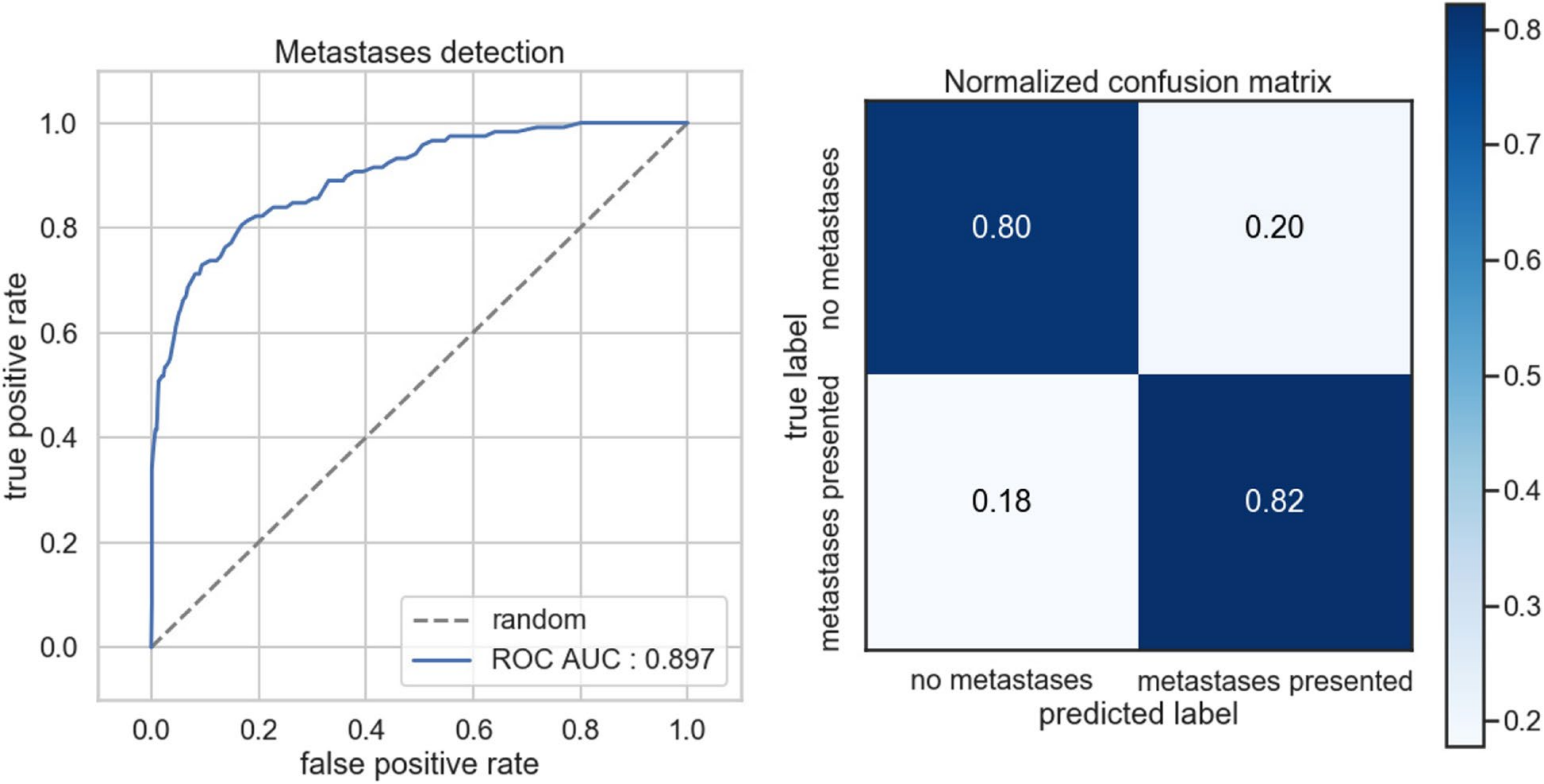
ROC curve for the classification DL model (left) and Confusion matrix (right)

The classification performances of the DL model were evaluated on the external test set coming from Centre C, in terms of Area under the Curve (AUC). The AUC gives the diagnostic ability of a binary classifier to discriminate between true and false values, in this case metastatic and non-metastatic bone disease. Figure 4 (left) represents the ROC curve of the DL classification model, while Fig. 4 (right) is the confusion matrix, which reports the percentages of correct and incorrect classification for each class (metastatic and non-metastatic).

The model achieved an AUC of 0.897, TPR of 82.2%, TNR of 80.45%, FPR of 19.55% and FNR of 17.79% on the external test set (n = 998). The model achieved a CLAIM score of 64% (27 out of 42 items) and STARD of 50% (15 out of 30 items).

### Explainability of trained model based on activation maps

**Fig. 5:**
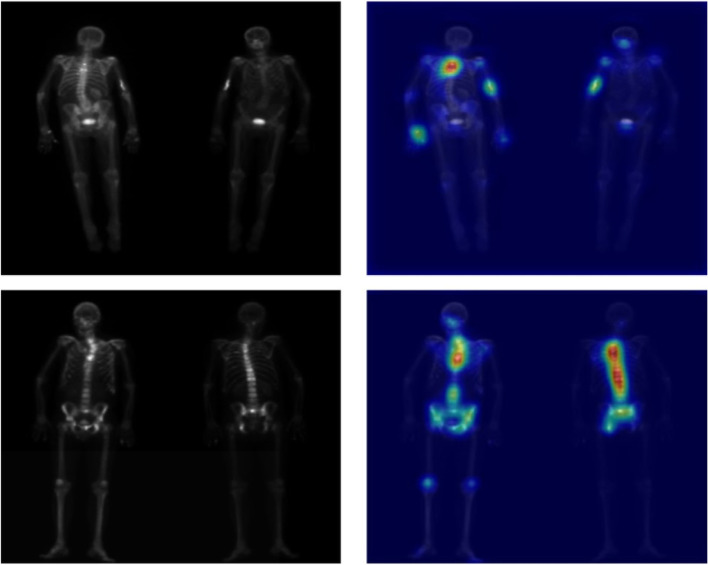
BS images which are correctly classified along with their corresponding activation maps extracted using the GRAD-CAM method. Left) original BD scan, Right) Grad-CAM activation maps obtained from the DL model. Scan correctly classified with a probability of 0.78 (top) and 0.99 (bottom)

**Fig. 6:**
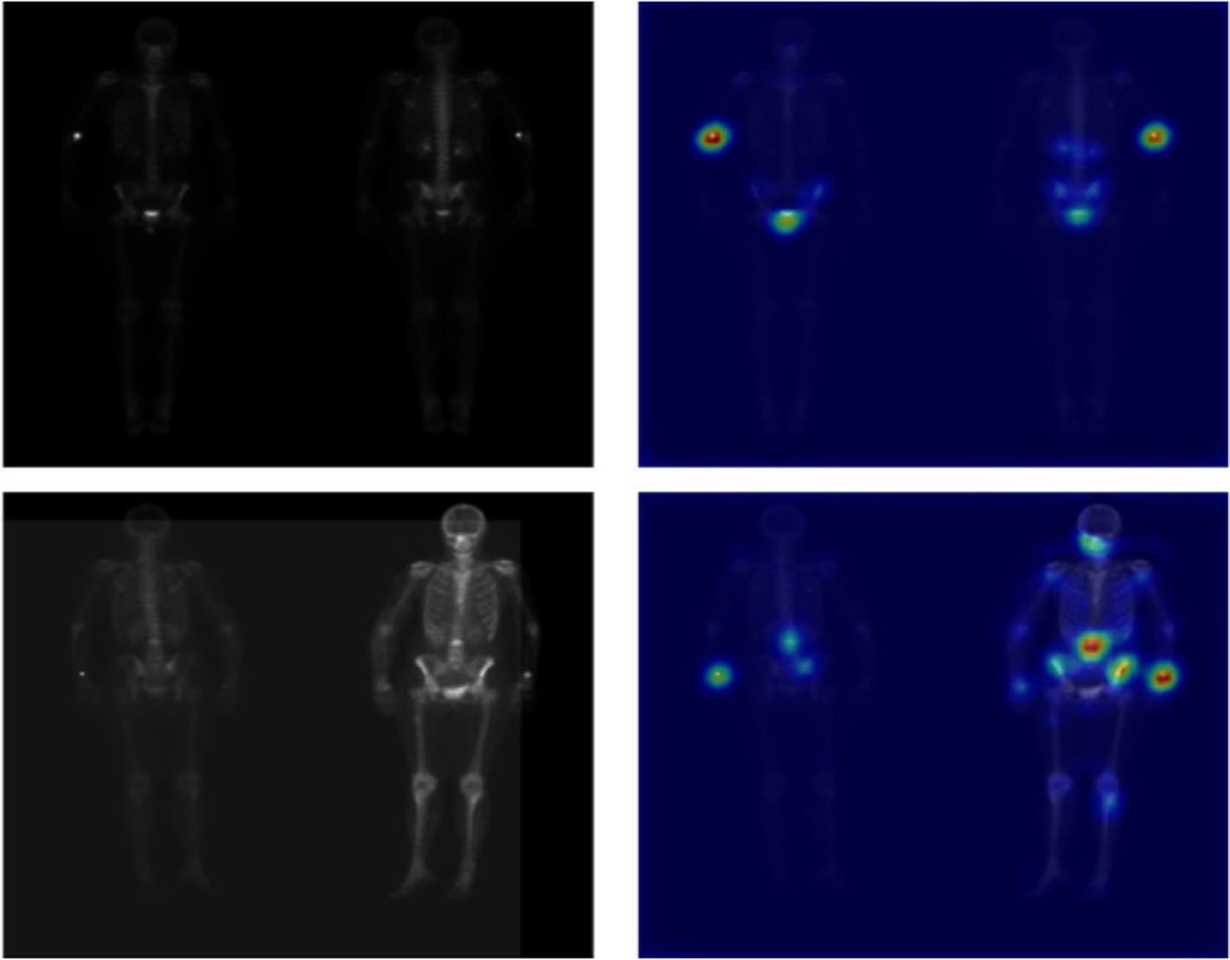
BS images which are wrongly classified along with their corresponding activation maps extracted using the GRAD-CAM method. Left) original BD scan, Right) Grad-CAM activation maps obtained from the DL model. Scan incorrectly classified with a probability of 0.79 (top) and 0.63 (bottom)

During the testing phase of the trained model, for the scans that were predicted positive (i.e. metastatic disease), activation maps were extracted using the Grad-CAM method. The method uses the gradients extracted corresponding to the class with highest predicted probability, flowing through the last convolutional layer, to produce the activation map. The map was then resized to the size of the input image and superimposed on the original BS scan, allowing visual inspection of activated zones on the image as shown in Figs. 5 and 6.

#### In silico clinical trial

**Fig. 7:**
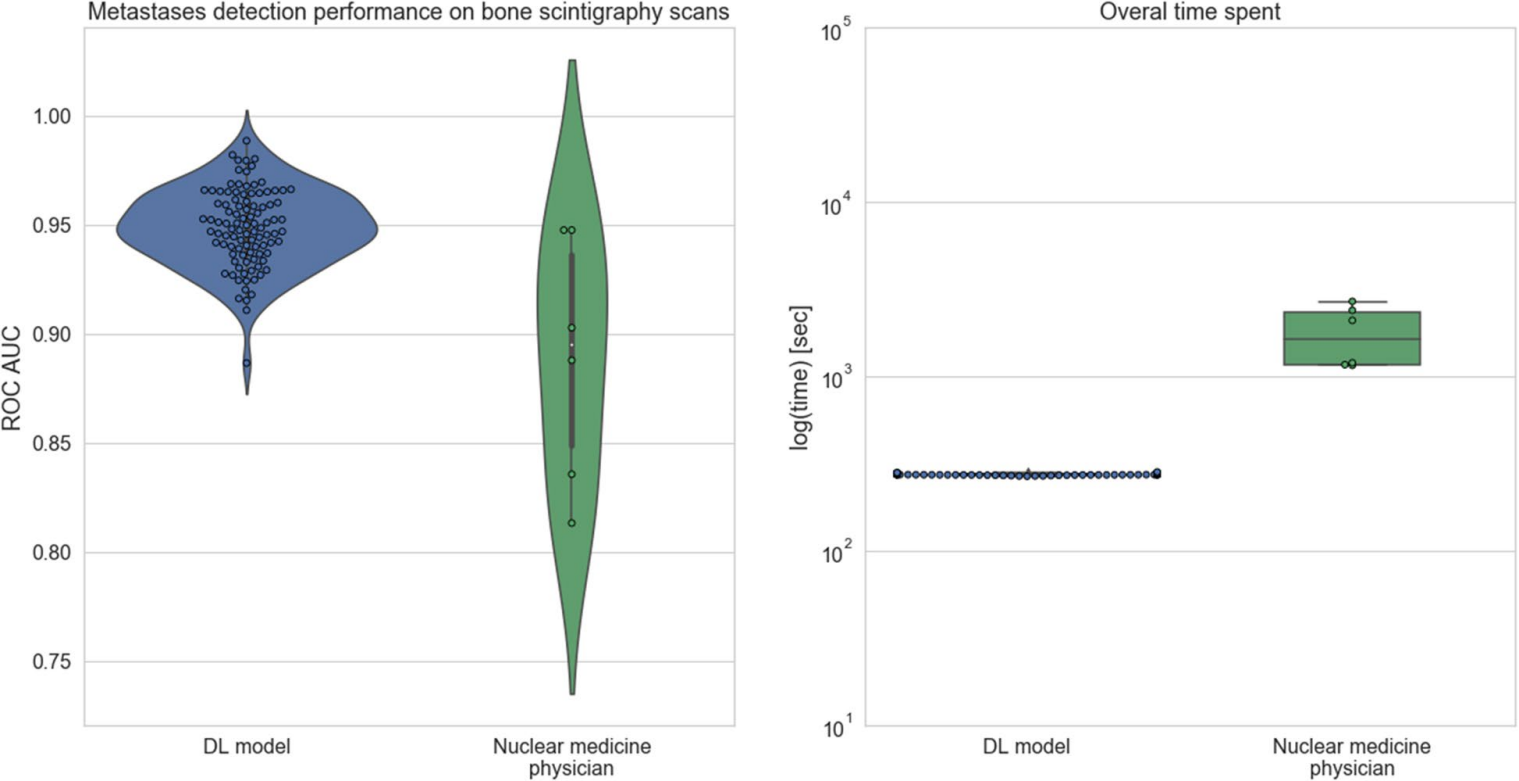
Violin plots showing the distributions of AUC scores for DL based and manual (across physicians) metastases detection on BS (left); boxplots of the log of the time needed by DL algorithm and nuclear medicine physicians (right)

The performance of nuclear medicine physicians based on the BS images was evaluated using AUC (Fig. 7, left), where median performance of the nuclear medicine physician was 0.895 (IQR = 0.087) with F1 score of 0.865 and median performance of DL based method was 0.95 (IQR = 0.024) with F1 score of 0.866.

On average, nuclear medicine physicians spent 30 min to classify all the 134 scans (Fig. 7, right). Given that the physicians had no access to clinical information about the patients, it takes on average 15 s to review one scan. In comparison, the developed algorithm takes 2 and half minutes to classify all the 134 scans, which is around 2 s per patient/ scan.

## Discussion

In this study, we investigated the potential of DL-based algorithms to detect MBD on BSs collected from different centres without limiting the study population to cancer patients. All BS scans were acquired at each center, following the standard of care, with different scanners brands and acquisition protocols, assuring the robustness and generalizability of the resulting DL model. Our results show that DL-based algorithms have a great potential to be applied as clinical decision aid tools, which could minimize the time needed by a nuclear physician to assess BSs, and increase the diagnostic specificity of BSs. The application of the state-of-the-art classification techniques has yielded a performance similar to nuclear physicians with no background about the patients’ history, which was further endorsed by the results of the in silico clinical trial.

Some studies previously investigated the potential of DL algorithms to classify lesions on BSs [27]. A study investigated the potential of a DL algorithm trained on 139 patients to detect MBD on BSs of prostate cancer patients [16]. The authors reported that the nuclear medicine physicians participating in the study achieved a higher sensitivity and specificity compared to the DL algorithm, though the differences were not statistically significant, and highlighted the possibility of involving DL in this clinical aspect. Another study also investigated the ability of DL algorithms to detect MBD in BS of prostate cancer patients [15]. However, the authors did not report on the comparison with the performance of nuclear medicine physicians. Another study investigated the performance of two DL architectures for classifying BS of prostate cancer patients [28]. The study included a large number of scans, and the authors reported that the best model achieved an overall accuracy of 0.9. Anand et al. reported on the performance of EXINI bone software, a classification tool for classifying BS of prostate cancer patients based on bone scan index, on simulated and patient scans [29]. The authors reported that the software was more consistent in classifying BS compared to visual assessment. Uniquely, we trained our model on patients with and without a history of cancer. The use of our developed algorithm resulted in better classification results on the external test set compared to the median nuclear medicine physician performance, in a significantly shorter time. These results highlight the potential of such algorithms to become reliable clinical decision support tools that minimize the time a clinician needs to review bone scintigraphy scans. Furthermore, Grad-CAM maps allow the nuclear physicians to rapidly check the spots based on which the classification was made. The activated regions are compared with radiologists’ segmentation of metastatic spots for qualitative assessment of the explainability of the model’s predictions on 25 BS scans (centre C) manually segmented by clinicians (Figs. 5 and 6). The activated regions superimposed on the image can be used in a clinical setting for qualitative assessment by radiologist which further impacts precise diagnosis. In the case of misclassification, Grad-CAM activation maps can help to quicky identify the area of the scan on which the model based its decision. In the reported case in Fig. 6, the image clearly evidence the injection spot located in the hand of the patients and other hyper intense regions in the pelvic bone as reasons for misclassification. This suggests the model which shows model’s overfitting [30] on features that are not relevant to the metastatic spot to classify presence or absence of metastasis in images.

While our study included a relatively large number of scans for training and externally testing the algorithm, several limitations of this study should be noted. Although explainability of model’s predictions were explored with qualitative assessment, this study lacks quantitative assessment of the activations due to the limited number of manual segmentations of metastasis (25) on the external test dataset. This could represent a strong point in the future development of the tool, with the availability of larger annotated datasets. Secondly, a prospective validation is required to properly assess the possible impact of the algorithm on the current standard of care, and considering other clinical characteristics of the patients (for example age, sex or primary tumour) that could influence classification performances. This is especially important given the current retrospective nature of the study, to prove beyond reasonable doubts that the classification performances are due to imaging features and not based on clinical/demographic data instead. Lastly, the physicians performances in the in silico trial are only indicative, as they were provided only with planar images, without corresponding SPECT and CT images, and without any clinical covariates available. Obviously, this approximates the actual routine in clinical settings, but it provides a fair indication of the potential added value of the proposed DL model.

## Conclusion

We developed a DL based algorithm that is able to detect MBD on BSs, with high specificity and sensitivity. This tool can be used also as a didactic support for radiologists in training. Further prospective validation is required before the algorithm can be used in the clinic

## Supplementary Information

**Additional file 1.**

## Data Availability

The data that support the findings of this study are not publicly available.

## Abbreviations

AP: Anteroposterior
AUC: Bone scintigraphy
DL: Deep learning
FNR: False negative rate
FPR: False positive rate
IQR: Interquartile range
MBD: Metastatic bone disease
PA: Posteroanterior
ROC: Receiver operating curve
TNR: True negative rate
TPR: True positive rate

## Declarations

Henry Woodruff has minority shares in the company Radiomics.

Felix M. Mottaghy received an advisor fee and reimbursement of travel costs from Radiomics. He reports institutional grants from GE and Nanomab outside the submitted work.

Mariaelena Occhipinti reports personal fees from Radiomics, outside the submitted work.

Wim Vos and Sean Walsh have shares in the company Radiomics.

## References

[1] Coleman RE (2006). Clinical features of metastatic bone disease and risk of skeletal morbidity. Clin cancer Res an Off J Am Assoc Cancer Res United States.

[2] Migliorini F, Maffulli N, Trivellas A, Eschweiler J, Tingart M, Driessen A. Bone metastases: a comprehensive review of the literature. Mol Biol Rep [Internet]. Department of Orthopaedics, University Clinic Aachen, RWTH Aachen University Clinic, Pauwelsstraße 30, 52074, Aachen, Germany. migliorini.md@gmail.com.; 2020;47:6337–45. Available from: http://europepmc.org/abstract/MED/32749632 10.1007/s11033-020-05684-0 32749632

[3] Huang J-F, Shen J, Li X, Rengan R, Silvestris N, Wang M et al. Incidence of patients with bone metastases at diagnosis of solid tumors in adults: a large population-based study. Ann Transl Med [Internet]. AME Publishing Company; 2020;8:482. Available from: https://pubmed.ncbi.nlm.nih.gov/32395526 10.21037/atm.2020.03.55 PMC7210217 32395526

[4] Coleman RE (2001). Metastatic bone disease: clinical features, pathophysiology and treatment strategies. Cancer Treat Rev Netherlands.

[5] Macedo F, Ladeira K, Pinho F, Saraiva N, Bonito N, Pinto L (2017). Bone metastases: an overview. Oncol Rev.

[6] Ryan PJ, Fogelman I (1997). Bone scintigraphy in metabolic bone disease. Semin Nucl Med United States.

[7] Ziessman HA, O’Malley JP, Thrall JHBT-NM, Fourth E, editors., editors. Chapter 7 - Skeletal Scintigraphy. Philadelphia: W.B. Saunders; 2014. p. 98–130. Available from: https://www.sciencedirect.com/science/article/pii/B9780323082990000079

[8] Van den Wyngaert T, Strobel K, Kampen WU, Kuwert T, van der Bruggen W, Mohan HK (2016). The EANM practice guidelines for bone scintigraphy. Eur J Nucl Med Mol Imaging.

[9] LeCun Y, Bengio Y, Hinton G. Deep learning. Nature [Internet]. 2015;521:436–44. Available from: 10.1038/nature14539 10.1038/nature14539 26017442

[10] McCulloch WS, Pitts W. A logical calculus of the ideas immanent in nervous activity. Bull Math Biophys [Internet]. 1943;5:115–33. Available from: 10.1007/BF02478259 2185863

[11] Deng L. A tutorial survey of architectures, algorithms, and applications for deep learning. APSIPA Trans Signal Inf Process [Internet]. 2014/01/22. Cambridge University Press; 2014;3:e2. Available from: https://www.cambridge.org/core/article/tutorial-survey-of-architectures-algorithms-and-applications-for-deep-learning/023B6ADF962FA37F8EC684B209E3DFAE

[12] Aslam YNS (2019). A Review of Deep Learning Approaches for Image Analysis. Int Conf Smart Syst Inven Technol..

[13] Janocha K, Czarnecki WM (2016). On loss functions for deep neural networks in classification. Schedae Informaticae.

[14] Cheng D.-C, Hsieh T.-C, Yen K.-Y, Kao C.-H. Lesion-Based Bone Metastasis Detection in Chest Bone Scintigraphy Images of Prostate Cancer Patients Using Pre-Train, Negative Mining, and Deep Learning. Diagnostics. 2021;11:518. 10.3390/diagnostics11030518. 10.3390/diagnostics11030518 PMC8000593 33803921

[15] Papandrianos, N.; Papageorgiou, E.; Anagnostis, A.; Papageorgiou, K. Efficient Bone Metastasis Diagnosis in Bone Scintigraphy Using a Fast Convolutional Neural Network Architecture. Diagnostics. 2020;10:532. 10.3390/diagnostics10080532. 10.3390/diagnostics10080532 PMC7459937 32751433

[16] Aoki Y, Nakayama M, Nomura K, Tomita Y, Nakajima K, Yamashina M (2020). The utility of a deep learning-based algorithm for bone scintigraphy in patient with prostate cancer. Ann Nucl Med Japan.

[17] Selvaraju RR, Cogswell M, Das A, Vedantam R, Parikh D, Batra D. Grad-CAM: Visual Explanations from Deep Networks via Gradient-Based Localization. Int J Comput Vis [Internet]. 2020;128:336–59. Available from: 10.1007/s11263-019-01228-7

[18] Dubost F, Adams H, Yilmaz P, Bortsova G, van Tulder G, Ikram MA et al. Weakly supervised object detection with 2D and 3D regression neural networks. Med Image Anal [Internet]. 2020;65:101767. Available from: https://www.sciencedirect.com/science/article/pii/S1361841520301316 10.1016/j.media.2020.101767 32674042

[19] Selvaraju RR, Cogswell M, Das A, Vedantam R, Parikh D, Batra D (2016). Grad-CAM: visual explanations from deep networks via gradient-based localization. Int J Comput Vis Springer.

[20] World Medical Association (2013). Declaration of Helsinki: ethical principles for medical research involving human subjects. JAMA United States.

[21] Simonyan, K. and Zisserman, A. (2015) Very Deep Convolutional Networks for Large-Scale Image Recognition. The 3rd International Conference on Learning Representations (ICLR2015). https://arxiv.org/abs/1409.1556.

[22] Shorten C, Khoshgoftaar TM. A survey on Image Data Augmentation for Deep Learning. J Big Data [Internet]. 2019;6:60. Available from: 10.1186/s40537-019-0197-0 10.1186/s40537-021-00492-0 PMC8287113 34306963

[23] Calin O, Activation Functions BT. - Deep Learning Architectures: A Mathematical Approach. In: Calin O, editor. Cham: Springer International Publishing; 2020. p. 21–39. Available from: 10.1007/978-3-030-36721-3_2

[24] Kingma, D. and Ba, J. (2015) Adam: A Method for Stochastic Optimization. Proceedings of the 3rd International Conference on Learning Representations (ICLR 2015). https://arxiv.org/abs/1412.6980.

[25] Mongan J, Moy L, Kahn CE. Checklist for Artificial Intelligence in Medical Imaging (CLAIM): A Guide for Authors and Reviewers. Radiol Artif Intell [Internet]. Radiological Society of North America; 2020;2:e200029. Available from: 10.1148/ryai.2020200029 10.1148/ryai.2020200029 PMC8017414 33937821

[26] Cohen JF, Korevaar DA, Altman DG, Bruns DE, Gatsonis CA, Hooft L et al. STARD 2015 guidelines for reporting diagnostic accuracy studies: explanation and elaboration. BMJ Open [Internet]. 2016;6:e012799. Available from: http://bmjopen.bmj.com/content/6/11/e012799.abstract 10.1136/bmjopen-2016-012799 PMC5128957 28137831

[27] Liu S, Feng M, Qiao T, Cai H, Xu K, Yu X (2022). Deep learning for the Automatic diagnosis and analysis of bone metastasis on bone scintigrams. Cancer Manag Res.

[28] Han S, Oh J.S, Lee J.J. Diagnostic performance of deep learning models for detecting bone metastasis on whole-body bone scan in prostate cancer. Eur J Nucl Med Mol Imaging. 2022;49:585–595. 10.1007/s00259-021-05481-2. 10.1007/s00259-021-05481-2 34363089

[29] Anand A, Morris MJ, Kaboteh R, Båth L, Sadik M, Gjertsson P (2016). Analytic Validation of the automated bone scan index as an imaging biomarker to standardize quantitative changes in bone scans of patients with metastatic prostate Cancer. J Nucl Med.

[30] Narasinga Rao MR, Venkatesh Prasad D, Sai Teja V, Zindavali P, Phanindra Reddy M (2018). A Survey on Prevention of Overfitting in Convolution neural networks using machine learning techniques. Int J Eng Technol.

