# Supplementary material for: Deep learning based identification of bone scintigraphies containing metastatic bone disease foci
Source: Cancer Imaging. 2023 Jan 25;23:12. doi: 10.1186/s40644-023-00524-3 (PMC9875407; doi:10.1186/s40644-023-00524-3)
Supplement: Supplementary file 1 — Additional file 1. [file 40644_2023_524_MOESM1_ESM.docx]

**Supplementary Information**

**Deep learning based identification of bone scintigraphies containing metastatic bone disease foci**

*Abdalla Ibrahim^1,2,3,4,*^, Akshayaa Vaidyanathan^1,5,*^, Sergey Primakov^1,2^, Flore Belmans^5^, Fabio Bottari^5^, Turkey Refaee^1,6^, Pierre Lovinfosse^3^, Alexandre Jadoul^3^, Celine Derwael^3^, Fabian Hertel^4^, Henry C. Woodruff^1,2^, Helle D. Zacho^7,8^, Sean Walsh^5^, Wim Vos^5^, Mariaelena Occhipinti^5^, François-Xavier Hanin^9^, Philippe Lambin^1,2,⇞^, Felix M. Mottaghy^2,4,⇞^, Roland Hustinx^3,⇞^*

** equal contribution*

*⇞ Senior authors contributed equally*

^1^ The D-Lab, Department of Precision Medicine, GROW- School for Oncology and Developmental Biology, Maastricht University, Maastricht, The Netherlands.

^2^ Department of Radiology and Nuclear Medicine, Maastricht University Medical Centre+, Maastricht, The Netherlands

^3^ Division of Nuclear Medicine and Oncological Imaging, Department of Medical Physics, Hospital Centre Universitaire De Liege, Liege, Belgium.

^4^ Department of Nuclear Medicine and Comprehensive diagnostic centre Aachen (CDCA), University Hospital RWTH Aachen University, Aachen, Germany

^5^ Radiomics (Oncoradiomics SA), Liege, Belgium

^6^ Department of Diagnostic Radiology, Faculty of Applied Medical Sciences, Jazan University, Jazan, Saudi Arabia.

^7^ Department of Nuclear Medicine, Clinical Cancer Research Centre, Aalborg University Hospital, Aalborg , Denmark

^8^ Department of Clinical Medicine, Aalborg University, Aalborg , Denmark

^9^ Department of Nuclear Medicine, Universite´ Catholique de Louvain, CHU-UCL-Namur, Belgium

**Keywords:**

Deep learning, Metastatic Bone Disease, Bone scintigraphy, Activation maps

**Corresponding author:**

Akshayaa Vaidyanathan

Radiomics (Oncoradiomics SA), Liege, Belgium

[akshayaa.vaidyanathan@radiomics.bio](mailto:akshayaa.vaidyanathan@radiomics.bio)

**CLAIM Checklist for Artificial Intelligence in Medical Imaging**

| **Section / Topic** | **No.** | **Item** |  |
| --- | --- | --- | --- |
| **TITLE** / **ABSTRACT** |  |  |  |
|  | **1** | Identification as a study of AI methodology, specifying the category of technology used (e.g., deep learning) | **x** |
|  | **2** | Structured summary of study design, methods, results, and conclusions | **x** |
| **INTRODUCTION** |  |  |  |
|  | **3** | Scientific and clinical background, including the intended use and clinical role of the AI approach | **x** |
|  | **4** | Study objectives and hypotheses | **x** |
| **METHODS** |  |  |  |
| ***Study Design*** | **5** | Prospective or retrospective study | **x** |
|  | **6** | Study goal, such as model creation, exploratory study, feasibility study, non-inferiority trial | **x** |
| ***Data*** | **7** | Data sources | **x** |
|  | **8** | Eligibility criteria: how, where, and when potentially eligible participants or studies were identified (e.g.,  symptoms, results from previous tests, inclusion in registry, patient-care setting, location, dates) | **x** |
|  | **9** | Data pre-processing steps | **x** |
|  | **10** | Selection of data subsets, if applicable | **x** |
|  | **11** | Definitions of data elements, with references to Common Data Elements | **x** |
|  | **12** | De-identification methods | **x** |
|  | **13** | How missing data were handled |  |
| ***Ground Truth*** | **14** | Definition of ground truth reference standard, in sufficient detail to allow replication | x |
|  | **15** | Rationale for choosing the reference standard (if alternatives exist) | **na** |
|  | **16** | Source of ground-truth annotations; qualifications and preparation of annotators | x |
|  | **17** | Annotation tools |  |
|  | **18** | Measurement of inter- and intra-rater variability; methods to mitigate variability and/or resolve discrepancies |  |
| ***Data Partitions*** | **19** | Intended sample size and how it was determined |  |
|  | **20** | How data were assigned to partitions; specify proportions | **x** |
|  | **21** | Level at which partitions are disjoint (e.g., image, study, patient, institution) | **x** |
| ***Model*** | **22** | Detailed description of model, including inputs, outputs, all intermediate layers and connections | **x** |
|  | **23** | Software libraries, frameworks, and packages | **x** |
|  | **24** | Initialization of model parameters (e.g., randomization, transfer learning) | **x** |
| ***Training*** | **25** | Details of training approach, including data augmentation, hyperparameters, number of models trained | **x** |
|  | **26** | Method of selecting the final model |  |
|  | **27** | Ensembling techniques, if applicable |  |
| ***Evaluation*** | **28** | Metrics of model performance | **x** |
|  | **29** | Statistical measures of significance and uncertainty (e.g., confidence intervals) | **x** |
|  | **30** | Robustness or sensitivity analysis |  |
|  | **31** | Methods for explainability or interpretability (e.g., saliency maps), and how they were validated | **x** |
|  | **32** | Validation or testing on external data | **x** |
| **RESULTS** |  |  |  |
| ***Data*** | **33** | Flow of participants or cases, using a diagram to indicate inclusion and exclusion |  |
|  | **34** | Demographic and clinical characteristics of cases in each partition |  |
| ***Model performance*** | **35** | Performance metrics for optimal model(s) on all data partitions |  |
|  | **36** | Estimates of diagnostic accuracy and their precision (such as 95% confidence intervals) | **x** |
|  | **37** | Failure analysis of incorrectly classified cases |  |
| **DISCUSSION** |  |  |  |
|  | **38** | Study limitations, including potential bias, statistical uncertainty, and generalizability | **x** |
|  | **39** | Implications for practice, including the intended use and/or clinical role | **x** |
| **OTHER INFORMATION** |  |  |  |
|  | **40** | Registration number and name of registry |  |
|  | **41** | Where the full study protocol can be accessed |  |
|  | **42** | Sources of funding and other support; role of funders | **x** |

**STARD - Standards for Reporting Diagnostic accuracy studies**

|  | **Section & Topic** | **No** | **Item** | **Reported on page #** |
| --- | --- | --- | --- | --- |
|  |  |  |  |  |
|  | **TITLE OR ABSTRACT** |  |  |  |
|  |  | **1** | Identification as a study of diagnostic accuracy using at least one measure of accuracy  (such as sensitivity, specificity, predictive values, or AUC) | 2 |
|  | **ABSTRACT** |  |  |  |
|  |  | **2** | Structured summary of study design, methods, results, and conclusions  (for specific guidance, see STARD for Abstracts) | 2 |
|  | **INTRODUCTION** |  |  |  |
|  |  | **3** | Scientific and clinical background, including the intended use and clinical role of the index test | 3 |
|  |  | **4** | Study objectives and hypotheses | 4 |
|  | **METHODS** |  |  |  |
|  | *Study design* | **5** | Whether data collection was planned before the index test and reference standard  were performed (prospective study) or after (retrospective study) | 5 |
|  | *Participants* | **6** | Eligibility criteria |  |
|  |  | **7** | On what basis potentially eligible participants were identified  (such as symptoms, results from previous tests, inclusion in registry) | 5 |
|  |  | **8** | Where and when potentially eligible participants were identified (setting, location and dates) | 5 |
|  |  | **9** | Whether participants formed a consecutive, random or convenience series |  |
|  | *Test methods* | **10a** | Index test, in sufficient detail to allow replication |  |
|  |  | **10b** | Reference standard, in sufficient detail to allow replication |  |
|  |  | **11** | Rationale for choosing the reference standard (if alternatives exist) |  |
|  |  | **12a** | Definition of and rationale for test positivity cut-offs or result categories  of the index test, distinguishing pre-specified from exploratory |  |
|  |  | **12b** | Definition of and rationale for test positivity cut-offs or result categories  of the reference standard, distinguishing pre-specified from exploratory |  |
|  |  | **13a** | Whether clinical information and reference standard results were available  to the performers/readers of the index test |  |
|  |  | **13b** | Whether clinical information and index test results were available  to the assessors of the reference standard |  |
|  | *Analysis* | **14** | Methods for estimating or comparing measures of diagnostic accuracy | 10 |
|  |  | **15** | How indeterminate index test or reference standard results were handled | 10 |
|  |  | **16** | How missing data on the index test and reference standard were handled |  |
|  |  | **17** | Any analyses of variability in diagnostic accuracy, distinguishing pre-specified from exploratory | 10 |
|  |  | **18** | Intended sample size and how it was determined |  |
|  | **RESULTS** |  |  |  |
|  | *Participants* | **19** | Flow of participants, using a diagram |  |
|  |  | **20** | Baseline demographic and clinical characteristics of participants |  |
|  |  | **21a** | Distribution of severity of disease in those with the target condition | 7 |
|  |  | **21b** | Distribution of alternative diagnoses in those without the target condition | 7 |
|  |  | **22** | Time interval and any clinical interventions between index test and reference standard |  |
|  | *Test results* | **23** | Cross tabulation of the index test results (or their distribution)  by the results of the reference standard |  |
|  |  | **24** | Estimates of diagnostic accuracy and their precision (such as 95% confidence intervals) | 12 |
|  |  | **25** | Any adverse events from performing the index test or the reference standard |  |
|  | **DISCUSSION** |  |  |  |
|  |  | **26** | Study limitations, including sources of potential bias, statistical uncertainty, and generalisability | 18-19 |
|  |  | **27** | Implications for practice, including the intended use and clinical role of the index test |  |
|  | **OTHER INFORMATION** |  |  |  |
|  |  | **28** | Registration number and name of registry |  |
|  |  | **29** | Where the full study protocol can be accessed |  |
|  |  | **30** | Sources of funding and other support; role of funders | 22 |
|  |  |  |  |  |
